# The effects and toxicity profiles of consolidative and salvage thoracic radiotherapy following chemoimmunotherapy in patients with extensive-stage small cell lung cancer

**DOI:** 10.7555/JBR.39.20250067

**Published:** 2025-05-27

**Authors:** Ruozhou Sun, Dan Zong, Xin Chen, Yizhi Ge, Ning Jiang, Lijun Zhao, Xue Song, Xia He, Xiangzhi Zhu

**Affiliations:** 1 Department of Radiation Oncology, the Affiliated Cancer Hospital of Nanjing Medical University, Jiangsu Cancer Hospital, Jiangsu Institute of Cancer Research, Nanjing, Jiangsu 210009, China; 2 Department of Environmental Genomics, Jiangsu Key Laboratory of Cancer Biomarkers, Prevention and Treatment, Collaborative Innovation Center for Cancer Personalized Medicine, Nanjing Medical University, Nanjing, Jiangsu 210009, China

**Keywords:** extensive-stage small cell lung cancer, thoracic radiotherapy, chemoimmunotherapy, survival rate, safety

## Abstract

The present study assessed the efficacy and safety of thoracic radiotherapy (TRT) following first-line chemotherapy or chemoimmunotherapy in patients with extensive-stage small cell lung cancer (ES-SCLC), focusing on the influence of different TRT timing strategies (consolidative *vs.* salvage) on survival rates. We retrospectively analyzed a total of 54 patients with ES-SCLC treated between January 2019 and July 2022. Patients receiving consolidative TRT (cTRT) within three months after completion of first-line treatment were compared with those receiving salvage TRT (sTRT) after disease progression. The primary endpoints were overall survival (OS), progression-free survival (PFS), locoregional-free survival (LRFS), and distant metastasis-free survival (DMFS); the secondary endpoint included safety. The cTRT group (*n* = 41) showed significantly longer median OS (26.6 *vs.* 14.8 months, *P* = 0.048), PFS (12.9 *vs.* 3.5 months, *P* < 0.0001), and DMFS (10.7 *vs.* 3.4 months, *P* = 0.0044) than the sTRT group (*n* = 13). Multivariate analysis revealed that cTRT was an independent, favorable prognostic factor. No significant differences in OS or LRFS were observed between high-dose (≥ 50 Gy) and low-dose (< 50 Gy) TRT. Hematologic and respiratory toxicities were the most frequently reported adverse events, with acceptable tolerability. In conclusion, cTRT after chemoimmunotherapy significantly improves survival outcomes for ES-SCLC patients, and low-dose TRT may be a suitable option.

## Introduction

Small cell lung cancer (SCLC) is among the most aggressive malignancies, comprising approximately 15% of all lung cancers^[1]^. SCLC is characterized by rapid proliferation, dysregulation of multiple signaling pathways, high vascularization, imbalanced apoptosis, and genetic instability^[2]^. About two-thirds of patients with SCLC have extrathoracic spread at the time of initial diagnosis, with a 5-year survival rate of only 7%^[3–4]^.

SCLC is categorized into limited and extensive stages according to the Veterans Administration Lung Study Group (VALSG) staging system^[5]^. For nearly 30 years, the standard first-line treatment for extensive-stage SCLC (ES-SCLC) has been platinum-based chemotherapy (carboplatin or cisplatin) combined with etoposide^[6]^. Despite the favorable response to initial chemotherapy, patients are highly prone to relapse, with a median survival of only 10 months^[7–8]^. The addition of an immune checkpoint inhibitor to platinum and etoposide has been approved by the FDA. Both the IMpower133 study^[9]^ and the CASPIAN study^[10]^ demonstrated that the combination of programmed cell death ligand 1 (PD-L1) inhibitor (atezolizumab or durvalumab) with platinum and etoposide could extend the overall survival (OS) of ES-SCLC patients by 2–3 months. Serplulimab in combination with chemotherapy also provided a significant survival advantage for ES-SCLC patients^[11]^. However, these randomized controlled studies have not addressed whether thoracic radiotherapy (TRT) may enhance patient survival or established the optimal timing for undergoing TRT.

In the era of immunotherapy, the efficacy of TRT in treating ES-SCLC remains controversial^[12–13]^. Preclinical studies have suggested a synergistic effect between radiotherapy and immunotherapy, highlighting that radiotherapy could modulate the immune system's interaction with tumor cells by activating immune cells and increasing exposure to tumor antigens^[14–15]^. The PACIFIC study showed that chemotherapy combined with durvalumab and TRT significantly prolonged progression-free survival (PFS) and OS in patients with unresectable stage Ⅲ non-SCLC, thereby establishing the foundation for the safety and efficacy of chemoimmunotherapy combined with radiotherapy^[16]^. However, the effectiveness and safety of chemoimmunotherapy combined with TRT in ES-SCLC patients remain uncertain. The optimal timing for TRT is a matter of ongoing debate and is not yet established. Therefore, the primary objective of the present study was to evaluate the efficacy and safety of TRT following first-line chemotherapy or chemoimmunotherapy in ES-SCLC patients, and to examine the effects of different TRT timing strategies (consolidative *vs.* salvage) on patient survival outcomes. By comparing consolidative TRT (cTRT) and salvage TRT (sTRT), we aimed to determine the optimal timing for TRT to maximize survival benefits in ES-SCLC patients.

## Materials and methods

### Patients

**Figure 1:**
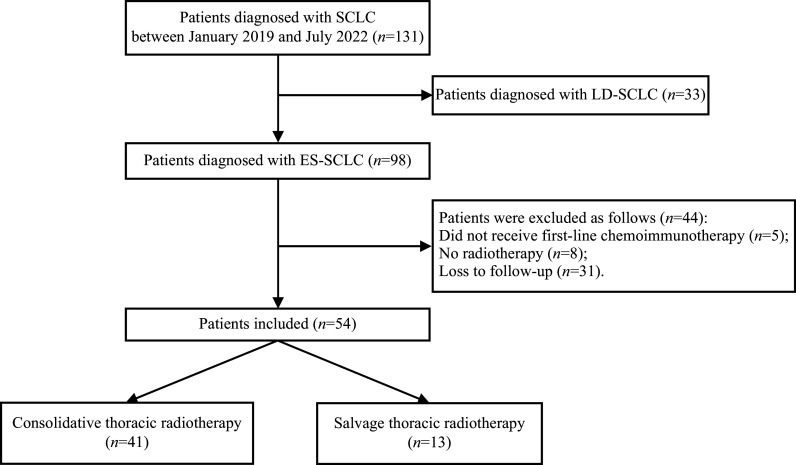
Diagram of the patient selection process. Diagram illustrating the selection process of patients diagnosed with small cell lung cancer (SCLC) between January 2019 and July 2022. Of 131 patients initially diagnosed, 33 were identified with limited-stage SCLC (LD-SCLC) and were excluded. The remaining 98 patients were diagnosed with extensive-stage SCLC (ES-SCLC). Among these, 44 patients were excluded due to not receiving first-line chemoimmunotherapy (*n* = 5), no radiotherapy (*n* = 8), or loss to follow-up (*n* = 31). Finally, 54 patients were included in the study, with 41 receiving consolidative thoracic radiotherapy and 13 receiving salvage thoracic radiotherapy.

We retrospectively analyzed patients diagnosed with SCLC at Nanjing Medical University Affiliated Cancer Hospital between January 2019 and July 2022. The primary inclusion criteria were as follows: (1) histological or cytological diagnosis of SCLC; (2) confirmed diagnosis of ES-SCLC according to the VALSG staging system; (3) all patients had received first-line platinum-containing chemotherapy regimens, with or without concurrent immunotherapy; and (4) patients underwent TRT. The exclusion criteria were as follows: (1) patients who did not receive first-line chemotherapy or chemoimmunotherapy; (2) patients who did not undergo TRT; or (3) patients who were lost to follow-up or had insufficient clinical data. Ultimately, 54 patients were included in the study. The patient selection process is shown in ***Fig. 1***.

### Chemotherapy and immunotherapy

All patients received chemotherapy with either etoposide (100 mg/m^2^, Days 1–3) and cisplatin (75 mg/m^2^, Day 1), or etoposide and carboplatin (AUC = 5) every 3 weeks for 4–6 cycles. Some patients also received concurrent immunotherapy, including either durvalumab (1500 mg) or atezolizumab (1200 mg) every 3 weeks, or either camrelizumab (200 mg) or tislelizumab (240 mg) every 2 weeks.

### TRT and grouping

All patients ultimately received TRT, either as consolidative treatment following first-line therapy or as salvage treatment upon disease progression. Notably, we classified the patients into two groups: those undergoing cTRT within three months after completing first-line treatment were categorized as the active group, while those undergoing sTRT following the progression of thoracic lesions were categorized as the passive group. TRT was administered using intensity-modulated radiotherapy. For treatment simulation, four-dimensional computed tomography was performed to evaluate respiratory-induced tumor motion and optimize target delineation. The gross tumor volume encompassed the post-chemotherapy residual tumor and involved lymph nodes, delineated based on pre-treatment imaging. To account for setup uncertainties and respiratory motion, the planning target volume was generated by expanding the gross tumor volume with an isotropic margin of 0.5–1.0 cm. To minimize the effects of respiratory motion and enhance treatment precision, respiratory management techniques were applied during both simulation and radiotherapy delivery. Depending on tumor location, motion amplitude, and individual patient tolerance, either active breathing control or respiratory gating was selectively employed. In the present study, cTRT was administered to eligible patients following the completion of first-line treatment, regardless of the presence of residual thoracic disease. The decision to administer cTRT was based on a multidisciplinary evaluation of disease status, treatment response, and patient condition. Prophylactic cranial irradiation was selectively performed at the discretion of the treating physician, considering treatment response and patient characteristics. Additionally, patients who developed brain metastases after initial treatment received therapeutic whole-brain radiotherapy.

### Follow-up assessments and endpoints

Patients were evaluated every two treatment cycles during chemoimmunotherapy and within one month after TRT. Follow-up visits were conducted every three months for the first two years after treatment, and every six months thereafter. Follow-up procedures included imaging tests (chest computed tomography [CT], brain magnetic resonance imaging, and neck ultrasound), laboratory tests (blood counts, liver and kidney function, and tumor markers), and clinical symptom records. The primary endpoints of the present study were OS, PFS, locoregional-free survival (LRFS), and distant metastasis-free survival (DMFS). OS was defined as the time from the initiation of treatment to death from any cause or the last follow-up, whichever occurred first. PFS was defined as the time from the initiation of treatment to disease progression or death from any cause, whichever occurred first. LRFS was defined as the time from the initiation of treatment to the first locoregional recurrence or death from any cause. DMFS was defined as the time from the initiation of treatment to the first distant metastasis or death from any cause. The secondary endpoint was the safety of the combined radiotherapy. Toxic effects were assessed according to the Common Terminology Criteria for Adverse Events (version 4.0). Tumor response to first-line therapy was measured by the Response Evaluation Criteria in Solid Tumors (RECIST) guidelines (version 1.1). The efficacy was categorized as complete response, partial response, stable disease, or progressive disease.

### Statistical analysis

All statistical analyses were performed using SPSS (version 27.0) and R software (version 4.3.3). The Kaplan–Meier method was employed to estimate patient OS, PFS, LRFS, and DMFS. The log-rank test was used to compare survival curves. The Cox proportional hazards model was utilized for univariate and multivariate analyses. Variables with *P* ≤ 0.1 in univariate analysis were considered candidate predictors. Additionally, clinically important covariates, including first-line treatment modality, TRT, maintenance therapy, and brain radiotherapy, were included in the multivariate Cox regression models to adjust for potential confounding effects, regardless of their univariate *P* values. The proportional hazards assumption was tested using Schoenfeld residuals. If the assumption was violated, a time-dependent covariate, *i.e.*, an interaction term with the natural logarithm of time [ln(time)], was introduced into the model. Two-tailed *P* < 0.05 was considered statistically significant. Post hoc power analysis showed that the study had sufficient power (> 80%) to detect medium-to-large effect sizes, consistent with the primary endpoints of the present study. Details are provided in ***Supplementary Table 1*** (available online).

## Results

### Patient characteristics

Between January 2019 and July 2022, we screened 54 eligible patients from a total of 131 SCLC patients admitted to the Nanjing Medical University Affiliated Cancer Hospital. The basic characteristics of the 54 ES-SCLC patients are shown in ***Table 1***. The median age was 65 years, and 81% of the patients were male. Twenty-nine (54%) patients received first-line chemoimmunotherapy, and 17 (31%) patients received maintenance immunotherapy. Among them, 14 patients (26%) had distant metastases at diagnosis, including seven cases of bone metastases, two cases of adrenal metastases, three cases of brain metastases, and two cases of liver metastases. A total of 21 (39%) patients received brain radiotherapy. Specifically, three patients underwent prophylactic cranial irradiation, while 18 patients received palliative brain radiotherapy because of brain metastases.

**Table 1:** Baseline characteristics of 54 ES-SCLC patients

Characteristics	cTRT (*n*=41)	sTRT (*n*=13)	*P*
Age [years; *n* (%)]			0.947
< 70	35 (85.4)	11 (84.6)	
≥70	6 (14.6)	2 (15.4)	
Sex [*n* (%)]			0.739
Male	33 (80.5)	11 (84.6)	
Female	8 (19.5)	2 (15.4)	
First-line treatment [*n* (%)]			0.198
Chemotherapy alone	21 (51.2)	4 (30.8)	
Chemoimmunotherapy	20 (48.8)	9 (69.2)	
Maintenance therapy [*n* (%)]			0.349
Yes	14 (34.1)	3 (23.1)	
No	27 (65.9)	10 (76.9)	
Brain radiotherapy [*n* (%)]			0.971
Prophylactic cranial irradiation	3 (7.3)	0	
Therapeutic cranial irradiation for brain metastases	13 (31.7)	5 (38.5)	
No cranial radiotherapy	25 (61.0)	8 (61.5)	
Metastatic sites at initial diagnosis [*n* (%)]			—
Bone metastases	2 (4.9)	5 (38.5)	
Brain metastases	3 (7.3)	0	
Adrenal metastases	2 (4.9)	0	
Liver metastases	1 (2.4)	1 (7.7)	
Data are presented as the number (percentage). Statistical comparisons between the cTRT (*n* = 41) and sTRT (*n* = 13) groups were performed using the Chi-square test or Fisher's exact test, as appropriate. *P*-values for metastatic sites were not calculated due to the small number of events. Abbreviations: cTRT, consolidative thoracic radiotherapy; sTRT, salvage thoracic radiotherapy.			

**Figure 2:**
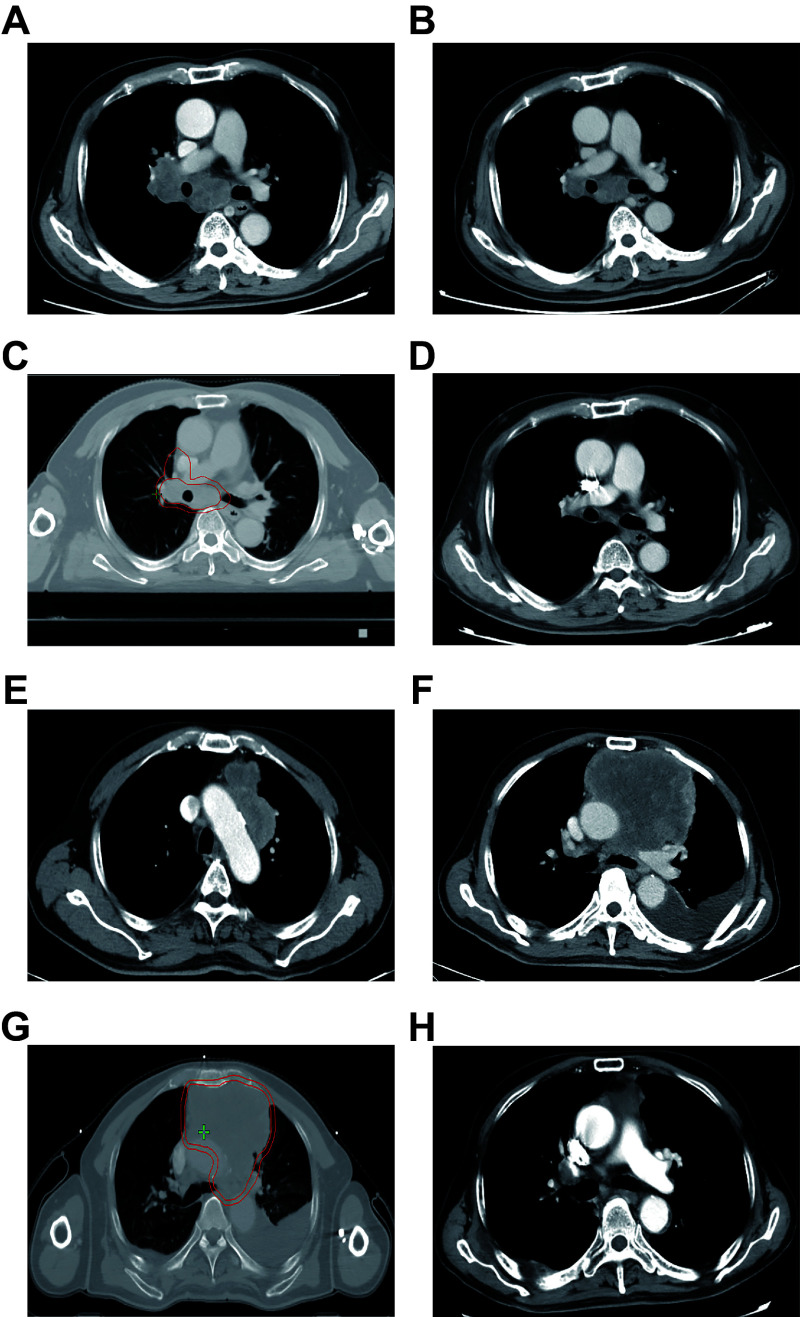
Computed tomography (CT) images and radiotherapy target volumes for patients in both the active and passive radiotherapy groups. A–D: CT images and radiotherapy target volumes for patients in the consolidative thoracic radiotherapy group are displayed as follows: pre-chemoimmunotherapy (A), pre-radiotherapy (B), radiotherapy target volume (C), and post-radiotherapy (D). E–H: For the salvage thoracic radiotherapy group, the corresponding images and volumes are shown as pre-chemoimmunotherapy (E), pre-radiotherapy (F), radiotherapy target volume (G), and post-radiotherapy (H).

All patients underwent TRT encompassing the primary site and the associated lymphatic drainage region. Based on the timing of radiotherapy application, 41 patients (76%) received cTRT and 13 patients (24%) received sTRT. The comparison of baseline characteristics between the cTRT and sTRT groups revealed no significant differences in age, sex, first-line treatment modality, maintenance therapy, brain metastases, or extent of metastatic disease, suggesting a relatively balanced distribution (***Table 1***). ***Fig. 2*** shows images of representative patients in the cTRT (***Fig. 2A***–***2D***) and sTRT (***Fig. 2E***–***2H***) groups, including pre-chemoimmunotherapy, pre-radiotherapy, radiotherapy target volume, and post-radiotherapy. The prescribed doses for TRT varied among the patients, with 60 Gy/30 fractions, 50 Gy/25 fractions, 45 Gy/15 fractions, and 30 Gy/10 fractions administered to 24% (13 cases), 6% (three cases), 37% (20 cases), and 33% (18 cases) of the patients, respectively.

### Survival analysis

The final follow-up was on October 30, 2023. The median follow-up time was 37.9 months (95% confidence interval [CI]: 33.7–42.2 months). Within the entire cohort, the median OS was 25.5 months (95% CI: 18.7–32.2 months), with corresponding 6-month, 1-year, and 2-year OS rates of 94.4%, 85.5%, and 50.3%, respectively (*Supplementary Fig. 1A*, available online). The median PFS was 8.6 months (95% CI: 7.6–9.5 months), with corresponding 6-month, 1-year, and 2-year PFS rates of 73.8%, 36.7%, and 23.3%, respectively (*Supplementary Fig. 1B*, available online).

**Figure 3:**
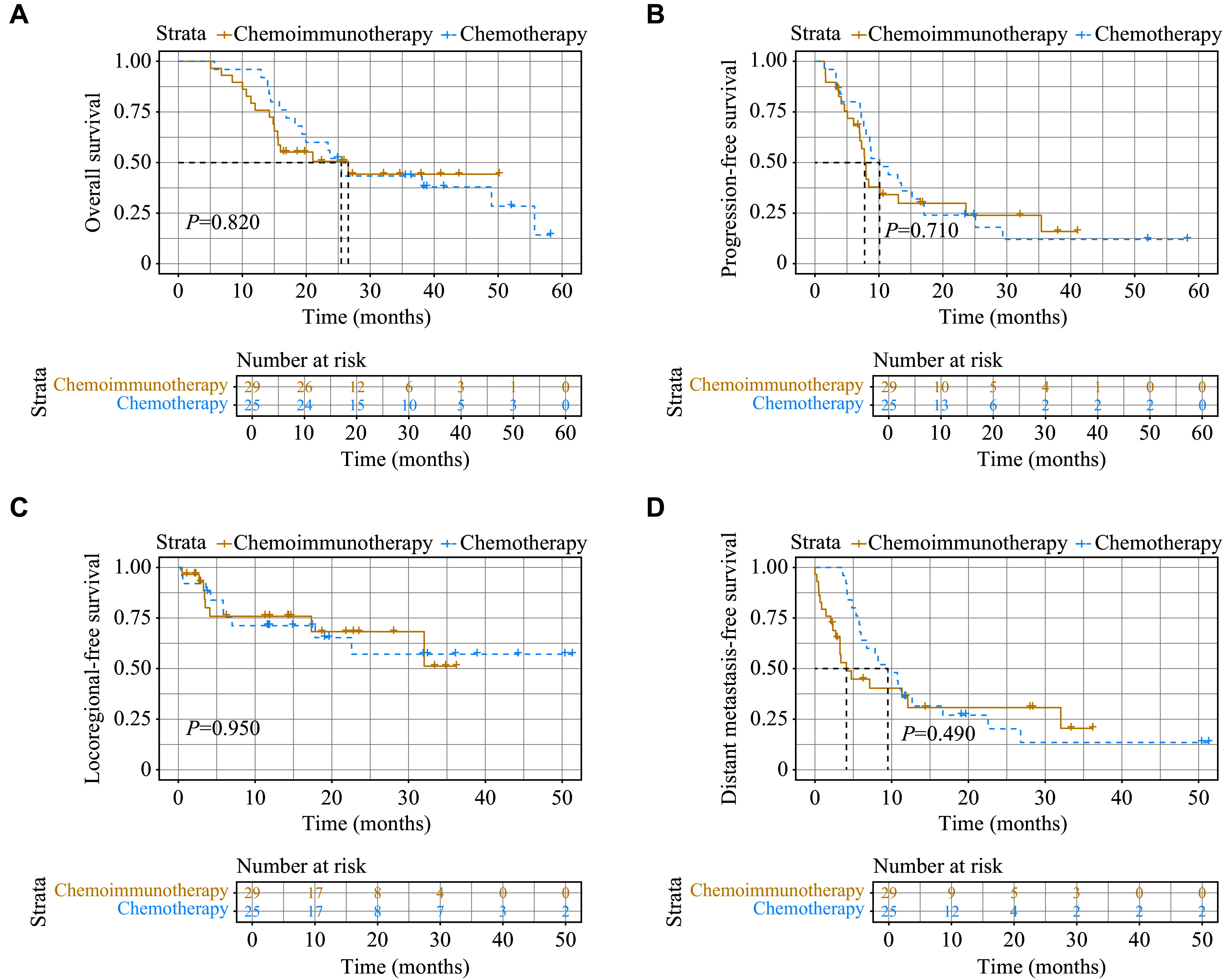
Kaplan–Meier curves comparing the survival outcomes between the chemoimmunotherapy and chemotherapy groups. A–D: The comparisons of survival outcomes for overall survival (A), progression-free survival (B), locoregional-free survival (C), and distant metastasis-free survival (D) between the two groups.

After the completion of first-line chemoimmunotherapy or chemotherapy, we assessed the therapeutic response using the RECIST guidelines, which included both the primary tumor and measurable metastatic lesions. Among the patients, 4% achieved a complete response, 67% had a partial response, 22% had progressive disease, and 7% had stable disease. The median OS of patients who received chemotherapy alone was 25.5 months (95% CI: 19.3 months to not applicable), and the median PFS was 10.1 months (95% CI: 8.0–17.1 months). For those who underwent chemoimmunotherapy, the median OS was 26.6 months (95% CI: 15.5 months to not applicable), and the median PFS was 7.8 months (95% CI: 7.0–23.6 months). Nevertheless, there were no significant differences in OS and PFS between the two groups (***Fig. 3A*** and ***3B***). Additionally, no significant differences were observed in the median LRFS (median not reached in both groups, *P* = 0.95) and median DMFS (9.5 *vs*. 4.1 months, *P* = 0.49) between the two groups (***Fig. 3C*** and ***3D***).

**Figure 4:**
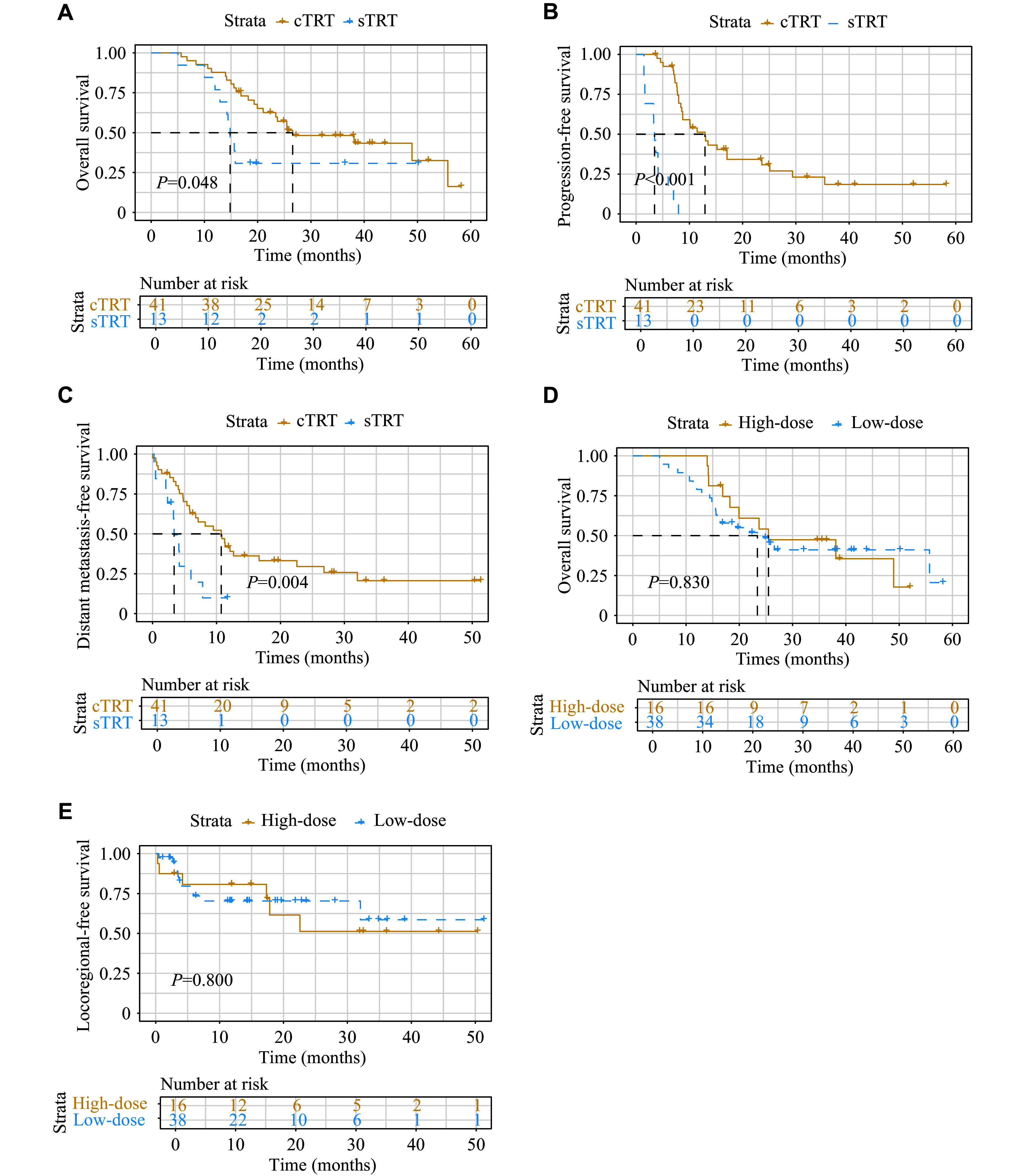
Kaplan–Meier curves illustrating survival outcomes in different treatment comparisons. A–D: The comparisons of survival outcomes for overall survival (A), progression-free survival (B), and distant metastasis-free survival (C) between the consolidative thoracic radiotherapy (cTRT) and salvage thoracic radiotherapy (sTRT) groups. D and E: Comparisons are made between the high-dose and low-dose groups for overall survival (D) and locoregional-free survival (E).

Furthermore, we performed subgroup analyses of consolidative and salvage TRT. Patients who received cTRT had a significantly longer median OS (26.6 *vs*. 14.8 months, *P* = 0.048) and PFS (12.9 *vs.* 3.5 months, *P* < 0.001) than those who underwent sTRT (***Fig. 4A*** and ***4B***). We also analyzed distant metastasis after radiotherapy in both groups, and found that patients receiving cTRT had a significantly longer median DMFS than those in the sTRT group (10.7 *vs.* 3.4 months, *P* = 0.004) (***Fig. 4C***). Additionally, we categorized the radiotherapy doses into high-dose and low-dose groups. However, high-dose radiotherapy did not show a significant survival advantage over low-dose radiotherapy (***Fig. 4D*** and ***4E***).

To further identify which variables predominantly influence survival outcomes, we performed univariate and multivariate Cox regression analyses (***Tables 2*** and ***3***). We assessed the associations between clinical characteristics (including age, sex, first-line treatment, TRT, maintenance therapy, and brain radiotherapy) and survival endpoints (OS, PFS, LRFS, and DMFS). In the univariate analysis, cTRT was significantly associated with improved OS (*P* = 0.05), PFS (*P* < 0.001), and DMFS (*P* = 0.006). Maintenance therapy was also significantly associated with better OS (*P* = 0.01). Other variables, including age, sex, first-line treatment regimen, and brain radiotherapy, showed no significant associations. Subsequently, variables with *P* ≤ 0.1 in the univariate analysis (TRT and maintenance therapy), as well as clinically relevant factors and known confounders (first-line treatment and brain radiotherapy), were included in the multivariate Cox regression models for OS, PFS, LRFS, and DMFS. The proportional hazards assumption was tested using Schoenfeld residuals, and a time-dependent covariate, *i.e.*, an interaction term with the natural logarithm of time [ln(time)], was added for maintenance therapy because of a violation of the assumption. Multivariate analysis demonstrated that cTRT was independently associated with improved OS (*P* = 0.05), PFS (*P* < 0.001), and DMFS (*P* = 0.005). After adjustment for the time interaction term, maintenance therapy remained significantly associated with better OS (*P* = 0.01), while its associations with other survival endpoints were not significant.

**Table 2:** Univariate analysis of survival in 54 EC-SCLC patients

Variables	OS			PFS			LRFS			DMFS	
	HR (95% CI)	*P*		HR (95% CI)	*P*		HR (95% CI)	*P*		HR (95% CI)	*P*
Age (years; ≥70 *vs.* < 70)	1.82 (0.74–4.50)	0.19		0.48 (0.17–1.35)	0.17		0.82 (0.19–3.61)	0.80		0.53 (0.19–1.50)	0.23
Sex (male *vs.* female)	1.82 (0.63–5.24)	0.27		1.07 (0.47–2.41)	0.88		0.82 (0.27–2.52)	0.73		0.92 (0.42–2.01)	0.84
First-line treatment (chemoimmunotherapy *vs.* chemotherapy)	1.09 (0.53–2.22)	0.82		1.12 (0.61–2.07)	0.71		0.97 (0.37–2.52)	0.95		1.25 (0.66–2.36)	0.49
Thoracic radiotherapy (cTRT *vs.* sTRT)	0.46 (0.21–1.01)	**0.05**		0.06 (0.03–0.16)	**< 0.001**		0.66 (0.21–2.06)	0.48		0.34 (0.16–0.74)	**0.006**
Maintenance therapy (yes *vs.* no)	0.32 (0.13–0.79)	**0.01**		0.91 (0.48–1.72)	0.78		0.76 (0.28–2.07)	0.59		0.69 (0.35–1.35)	0.28
Brain radiotherapy (yes *vs.* no)	1.40 (0.69–2.83)	0.36		1.14 (0.62–2.12)	0.67		0.90 (0.33–2.44)	0.84		1.32 (0.69–2.52)	0.40
Univariate Cox proportional hazards models were used to evaluate associations between clinical variables and survival outcomes, including OS, PFS, LRFS, and DMFS. HRs, 95% CIs, and *P*-values are reported. *n* = 54. Bold fonts indicate *P* ≤ 0.05.Abbreviations: CI, confidence interval; cTRT, consolidative thoracic radiotherapy; DMFS, distant metastasis-free survival; HR, hazard ratio; LRFS, locoregional-free survival; OS, overall survival; PFS, progression-free survival; sTRT, salvage thoracic radiotherapy.											

**Table 3:** Multivariate analysis of survival in 54 EC-SCLC patients

Variables	OS			PFS			LRFS			DMFS	
	HR (95% CI)	*P*		HR (95% CI)	*P*		HR (95% CI)	*P*		HR (95% CI)	*P*
First-line treatment (chemoimmunotherapy *vs.* chemotherapy)	1.13 (0.52–2.43)	0.75		0.76 (0.38–1.51)	0.43		0.97 (0.36–2.57)	0.95		1.44 (0.75–2.78)	0.27
Thoracic radiotherapy (cTRT *vs.* sTRT)	0.43 (0.18–1.02)	**0.05**		0.06 (0.02–0.15)	**< 0.001**		0.64 (0.20–2.04)	0.45		0.32 (0.15–0.71)	**0.005**
Maintenance therapy (yes *vs.* no)	0.30 (0.11–0.77)	**0.01**		0.97 (0.47–1.98)	0.93		0.71 (0.24–2.03)	0.52		0.65 (0.31–1.36)	0.25
Brain radiotherapy (yes *vs.* no)	1.07 (0.52–2.23)	0.84		1.15 (0.58–2.29)	0.69		0.83 (0.30–2.34)	0.73		1.14 (0.57–2.30)	0.71
Multivariate Cox proportional hazards models were used to identify factors independently associated with survival outcomes (OS, PFS, LRFS, and DMFS). Maintenance therapy was modeled as a time-dependent covariate because of a violation of the proportional hazards assumption. Adjusted HRs, 95% CIs, and *P*-values are presented. *n* = 54. Bold fonts indicate *P* ≤ 0.05.Abbreviations: CI, confidence interval; cTRT, consolidative thoracic radiotherapy; DMFS, distant metastasis-free survival; HR, hazard ratio; LRFS, locoregional-free survival; OS, overall survival; PFS, progression-free survival; sTRT, salvage thoracic radiotherapy.											

### Safety evaluation

Treatment-related adverse events are summarized in ***Table 4***. Two patients did not complete radiotherapy because of myelosuppression and esophagogastric reactions. The most frequently observed adverse effects were hematologic and respiratory toxicities. The most common hematologic toxicities included anemia (87%), leukopenia (78%), and thrombocytopenia (48%), with severity ranging from grade 1 to 4. Respiratory toxicities had the highest incidence of pneumonitis (46%), followed by cough (30%), dyspnea (23%), and fever (26%), with severity ranging from grade 1 to 2. Gastrointestinal toxicities included esophagitis, odynophagia, decreased appetite, and elevated transaminase levels. The occurrence ranged from 13% to 21%, with severity from grade 1 to 3. Additionally, some patients experienced systemic discomfort, such as fatigue (24%) and skin reactions (9%), with severity ranging from grade 1 to 3. Some significant biochemical indicators, including changes in lactate dehydrogenase and cardiac enzymes, were also investigated in the current study, with occurrences of 67% and 34%, respectively, and severity ranging from grade 1 to 4. Importantly, there were no grade 5 treatment-related adverse events.

**Table 4:** Summary of the adverse events during treatment

Toxicity	G1	G2	G3	G4
Hematologic				
Leukopenia	2 (4%)	18 (33%)	13 (24%)	9 (17%)
Anemia	18 (33%)	22 (41%)	5 (9%)	2 (4%)
Thrombocytopenia	13 (24%)	5 (9%)	5 (9%)	3 (6%)
Respiratory				
Pneumonitis	12 (22%)	13 (24%)	0	0
Cough	10 (19%)	6 (11%)	0	0
Dyspnea	9 (17%)	3 (6%)	0	0
Fever	3 (6%)	11 (20%)	0	0
Gastrointestinal				
Esophagitis	1 (2%)	5 (9%)	2 (4%)	0
Odynophagia	2 (4%)	4 (7%)	1 (2%)	0
Decreased appetite	8 (15%)	2 (4%)	1 (2%)	0
Elevated transaminase	10 (19%)	1 (2%)	0	0
Systemic disease				
Fatigue	7 (13%)	4 (7%)	2 (4%)	0
Skin reaction	4 (7%)	1 (2%)	0	0
Others				
Lactate dehydrogenase	25 (46%)	8 (15%)	2 (4%)	1 (2%)
Cardiac enzymes	10 (19%)	7 (13%)	0	1 (2%)
Adverse events are reported as the number (percentage) and categorized by grade (G1–G4) according to the Common Terminology Criteria for Adverse Events (version 4.0). Toxicities are grouped into hematologic, respiratory, gastrointestinal, systemic disease, and other categories. *n* = 54.				

## Discussion

In the present retrospective study, we evaluated the efficacy and safety of TRT following first-line chemoimmunotherapy or chemotherapy in ES-SCLC patients. Our findings indicate that patients who received cTRT had significantly prolonged OS, PFS, and DMFS compared with those who received sTRT. Furthermore, multivariate analysis identified cTRT as an independent favorable prognostic factor for OS, PFS, and DMFS, highlighting its potential role in optimizing the timing of TRT to improve long-term outcomes for ES-SCLC patients.

Although immunotherapy has shown promise in ES-SCLC, its efficacy is influenced by multiple factors, including the patient's overall condition, tumor characteristics, and treatment regimen^[17]^. In the present study, the chemotherapy-alone group had a median OS of 25.5 months and a median PFS of 10.1 months, while the chemoimmunotherapy group showed a comparable median OS (26.6 months) but shorter median PFS (7.8 months). Notably, our analysis revealed no significant differences in OS, PFS, LRFS, or DMFS between the chemoimmunotherapy and chemotherapy-alone groups, suggesting that the addition of immunotherapy did not provide a substantial survival advantage in this cohort.

Our results showed that patients in the cTRT group had a significantly extended median OS (26.6 *vs.* 14.8 months, *P* = 0.048), PFS (12.9 *vs.* 3.5 months, *P* < 0.0001), and DMFS (10.7 *vs.* 3.4 months, *P* = 0.0044), compared with the sTRT group. These findings are consistent with previous studies, including Jeremic *et al*^[18]^, who first demonstrated improved OS with TRT after chemotherapy in ES-SCLC. Univariate analysis revealed significant associations of cTRT with OS, PFS, and DMFS, while multivariate analysis demonstrated cTRT as an independent favorable prognostic factor (all *P* < 0.05). The high proportion of patients receiving cTRT (76%) may partly explain our better survival outcomes compared with previous reports. Similarly, the CREST study found TRT to be particularly beneficial in patients with residual thoracic disease and limited metastases^[19–20]^.

The optimal dose of TRT remains debated. Xu *et al*^[21]^ demonstrated that TRT > 50 Gy significantly improved 2-year OS and local control. Conversely, Han *et al*^[22]^ found that hyperfractionated radiotherapy (45 Gy/30 fractions, twice daily) offered better OS (22.7 *vs.* 18.2 months, *P* = 0.036) and PFS (11.3 *vs.* 9.3 months, *P* = 0.047) than conventional fractionation (60 Gy/30 fractions). Patients who received TRT within six chemotherapy cycles also had longer LRFS. Liu *et al*^[23]^ demonstrated that palliative hypofractionated TRT combined with first-line chemoimmunotherapy prolonged PFS, supporting alternative TRT regimens. Recently, the RISE study divided ES-SCLC patients into three groups to evaluate dose-escalated chest RT (45 Gy/15 fractions) plus SBRT (24 Gy/3 fractions) for up to 10 metastatic lesions, with the aim of informing optimal RT integration in ES-SCLC^[24]^. In the current study, we compared different prescribed TRT doses, including 60 Gy/30 fractions, 50 Gy/25 fractions, 45 Gy/15 fractions, and 30 Gy/10 fractions. Notably, we found no significant differences in OS or LRFS between patients receiving high-dose (≥ 50 Gy) and low-dose (< 50 Gy) TRT, suggesting that lower-dose regimens may be a viable alternative. These findings warrant further investigation, as reducing the radiation dose can minimize treatment-related toxicity while maintaining therapeutic efficacy.

In addition to TRT parameters, maintenance therapy emerged as a significant prognostic factor in the current study. Univariate analysis indicated that maintenance therapy was significantly associated with OS, while multivariate analysis demonstrated it as an independent and favorable prognostic factor. Given these results, maintenance therapy should be further emphasized in ES-SCLC treatment strategies, as it may play a critical role in improving long-term survival outcomes.

Immunotherapy, which carries specific toxicity risks, remains a cautious option when combined with TRT. The safety profile of concurrent or sequential TRT with immunotherapy in ES-SCLC remains underexplored. A phase Ⅰ clinical trial by Welsh *et al*^[25]^ demonstrated that pembrolizumab combined with TRT, followed by induction chemotherapy, was well tolerated, with no grade 4 to 5 adverse events reported. Similarly, Diamond *et al*^[26]^ evaluated the safety and efficacy of cTRT following first-line chemoimmunotherapy, reporting a median OS of 16 months, a 6-month OS rate of 94.7%, and a 12-month OS rate of 77.5%, with an acceptable safety profile (only 5% developed grade 2 esophagitis). Our findings also support the overall safety of TRT after chemoimmunotherapy, with no grade 5 toxicities observed. Hematologic and respiratory toxicities were the most common adverse effects in our study, but they were largely manageable. The incidence of anemia (87%) and leukopenia (78%) aligned with prior reports, while radiation pneumonitis (46%) remained a primary concern. This relatively high pneumonitis rate suggests a potential interaction between radiotherapy and immunotherapy, warranting careful monitoring^[27]^.

The current study has several limitations. First, as a retrospective study, potential selection bias cannot be excluded. The absence of baseline disease severity indicators, such as gross tumor volume, may have affected survival outcomes and limited comparisons between the cTRT and sTRT groups. Second, heterogeneity in radiotherapy regimens and post-progression treatments may have introduced confounding factors. Third, a substantial proportion of patients were lost to follow-up, potentially underestimating long-term survival benefits and treatment-related toxicities. In our upcoming prospective study, we will systematically address these limitations and improve future analyses.

In conclusion, adding cTRT following the completion of first-line chemotherapy combined with immunotherapy may improve the prognosis of ES-SCLC patients. Additionally, emphasizing maintenance therapy in treatment strategies is crucial. Low-dose TRT may be adopted, with treatment-related adverse events being well tolerated and clinically manageable.

## SUPPLEMENTARY DATA

Supplementary data to this article can be found online.
