# Supplementary material for: The effects and toxicity profiles of consolidative and salvage thoracic radiotherapy following chemoimmunotherapy in patients with extensive-stage small cell lung cancer
Source: J Biomed Res. 2025 May 27;39(5):467–77. doi: 10.7555/JBR.39.20250067 (PMC12481674; doi:10.7555/JBR.39.20250067)
Supplement: Supplementary file 1 — Supplementary data to this article can be found online. [file jbr-39-5-467-Supplementary.pdf]

Available online at www.jbr-pub.org.cn

Open Access at PubMed Central

Journal of Biomedical Research, 2025 39(5): 1–2

JBR

Supplementary Data

The effects and toxicity profiles of consolidative and salvage thoracic radiotherapy following chemoimmunotherapy in patients with extensive-stage small cell lung cancer

Ruo-zhou Sun[1,2,△], Dan Zong[1,2,△], Xin Chen[1,2], Yizhi Ge[1], Ning Jiang[1], Lijun Zhao[1], Xue Song[1], Xia He[1,2,✉], Xiangzhi Zhu[1,✉]

[1]Department of Radiation Oncology, the Affiliated Cancer Hospital of Nanjing Medical University, Jiangsu Cancer Hospital, Jiangsu Institute of Cancer Research, Nanjing, Jiangsu 210009, China;
[2]Department of Environmental Genomics, Jiangsu Key Laboratory of Cancer Biomarkers, Prevention and Treatment, Collaborative Innovation Center for Cancer Personalized Medicine, Nanjing Medical University, Nanjing, Jiangsu 210009, China.

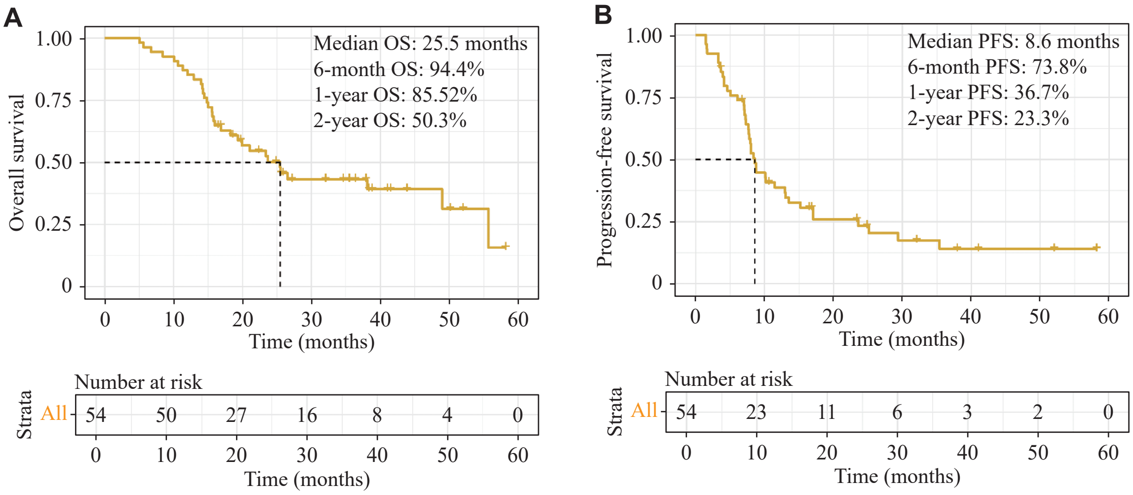

Supplementary Fig. 1 **Kaplan–Meier curves depicting (A) overall survival, (B) progression-free survival for the entire cohort.** Abbreviations: OS, overall survival; PFS, progression-free survival.

△These authors contributed equally to this work.

✉Corresponding authors: Xia He and Xiangzhi Zhu, Department of Radiation Oncology, the Affiliated Cancer Hospital of Nanjing Medical University, Jiangsu Cancer Hospital, Jiangsu Institute of Cancer Research, 42 Baiziting Road, Nanjing, Jiangsu 210009, China. E-mail: hexiabm@163.com (He) and 13182948068@163.com (Zhu).

Received: 19 February 2025; Revised: 19 May 2025; Accepted: 22 May 2025; Published online: 27 May 2025

CLC number: R734.2, Document code: A

The authors reported no conflict of interests.

This is an open access article under the Creative Commons Attribution (CC BY 4.0) license, which permits others to distribute, remix, adapt and build upon this work, for commercial use, provided the original work is properly cited.

© 2025 by Journal of Biomedical Research.

https://doi.org/10.7555/JBR.39.20250067

Supplementary Table 1 **Statistical power for different effect sizes**

Effect size (Cramér's V)	Statistical power (%)	Interpretation
Small (V=0.1)	11.4	Insufficient for small effects
Medium (V=0.3)	59.7	Limited for medium effects
Large (V=0.5)	95.7	Sufficient for large effects

A post hoc power analysis was conducted based on the sample size (n = 54) and a significance level of 0.05. Statistical power for detecting effect sizes (Cramér's V) of 0.1, 0.3, and 0.5 was calculated using the Chi-square tests.
